# Fight for the People's Health: The Application of Al Multiagent Systems in Medical Consortia

**DOI:** 10.1002/hcs2.70037

**Published:** 2026-04-02

**Authors:** Zihao Bian, Zhiyi Luo, Wenhui Zhang, Fanyi Kong, Yiyi Yang, Yiyang Chen, Chenjia Liao, Ziyi Chen, Wei Wang, Wanyun Zhong, Tuo Li, Nan Wang, Rongfang Zhu, Gen Li, Kangwei Shi, Ruizhe Shi, Zeyu Zhang, Zongjiu Zhang

**Affiliations:** ^1^ Institute for Hospital Management, Shenzhen International Graduate School Tsinghua University Shenzhen China; ^2^ School of Clinical Medicine (Beijing Tsinghua Changgung Hospital) Tsinghua University Beijing China; ^3^ Hospital, Tsinghua University of Integrated Traditional Chinese and Western Medicine Tsinghua University Beijing China; ^4^ School of Management Hefei University of Technology Hefei China; ^5^ Key Laboratory of Philosophy and Social Sciences for Cyberspace Behaviour and Management Hefei China; ^6^ School of Information Management Nanjing University Nanjing China; ^7^ Jiangsu Key Laboratory of Data Engineering and Knowledge Service Nanjing China; ^8^ Medicine Tsinghua University Beijing China

**Keywords:** artificial intelligence, healthcare decision support, healthcare service process optimization, medical consortium, multiagent system, resource allocation

## Abstract

**Background:**

The nationwide implementation of medical consortia at both district and county levels has reshaped China's healthcare system profoundly by establishing collaborative institutional networks and tiered service delivery pathways. However, difficulties such as loose referral, fragmented information, and resource disparity have hampered the delivery of integrated care in these consortia. Leveraging cutting‐edge information technology, this study aims to propose a set of AI‐driven integrated medical alliance solutions catering to the needs of patients, medical workers, and administrators.

**Methods:**

In the study, we introduce a multiagent system using the coordinator worker model and role‐based architecture. The system uses the retrieval‐augmented generation (RAG) framework, the ERNIE model, the chain of thought (CoT) reasoning mechanism, and an interactive platform. It is capable of enhancing full life‐cycle healthcare service by supporting patients' navigation of the system, doctors' clinical decision‐making, and hospital management, providing key functions like triage guidance, medical research assistance, and real‐time hospital operational data analysis.

**Results:**

This intelligent medical decision support platform provides tailored healthcare, ensures treatment continuity, improves decision‐making quality, and optimizes resource allocation efficiency.

**Discussion:**

Following the detailed analysis of the applications and advantages of the framework, the study further explores the challenges faced during the implementation of this platform, particularly related to hallucination, data security, and cost control.

**Conclusions:**

Finally, it calls for continued efforts to build intelligent, equitable, and high‐value healthcare systems through expanded applications of medical multiagent systems.

## Graphical abstract

This study describes a multiagent system using the “orchestrator‐worker” model and role‐based architecture to create an interactive platform. The system draws on retrieval‐augmented generation, large language models, and the chain of thought (CoT) reasoning mechanism.

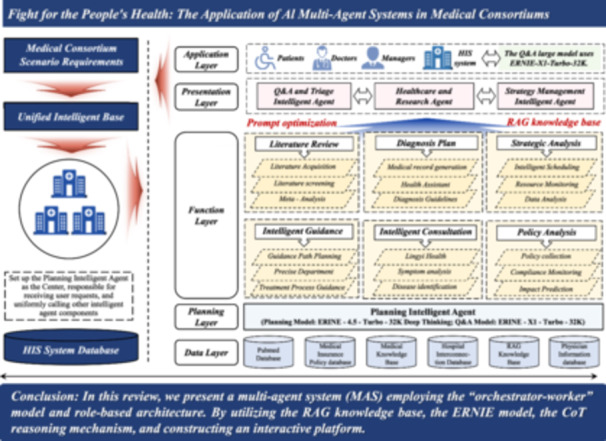

**Abbreviations**
API: application programming interface
CoT: chain of thought
DIP: diagnosis‐intervention
DRG: diagnosis‐related group
HIS: hospital information system
LLM: large language model
MAS: multiagent system
MC: medical consortium
RAG: retrieval‐augmented generation
SOAP: Subjective, Objective, Assessment, Plan

## 1 Background

Achieving an integrated healthcare system through structural optimization is a core element of the Healthy China strategy. The key institutional mechanism to address imbalanced resource distribution and fragmented services is the medical consortium (MC) model. This model has covered 2188 counties in China since its comprehensive implementation in 2017 [1, 2], with over 18,000 collaborative networks formed [3]. However, there are still coordination problems among medical providers, patients, and administrators. For example, patients often experience unclear referral pathway guidance, fragmented chronic disease care, and misunderstandings about health insurance policies. Doctors need to address inefficient knowledge retrieval and administrative tasks encroaching on clinical decision‐making time. Administrators urgently need intelligent tools to use within the diagnosis‐related group (DRG) and diagnosis‐intervention packet (DIP) healthcare payment reforms to allocate resources and provide quality control.

The MC model has provided institutional approaches to support the move to integrated care. However, it has also increased the urgency and difficulty of addressing these challenges. To reduce redundant and unnecessary expenses, consortia must shift the current disease‐centered service to a health‐centered mode by enhancing two‐way referrals, mutual recognition of medical results, and chronic condition management. To encourage more patients to use primary care, doctors and general practitioners at lower levels should improve their health management and clinical decision‐making abilities. Specialists from tertiary hospitals need more time to invest in the treatment and research of complex diseases. With a larger collaboration network, administrators in consortia are under significant pressure to dynamically coordinate cross‐institutional health resources, such as hospital beds and physicians, to remain efficient. These emerging demands indicate that traditional manual coordination mechanisms can no longer support the needs of complex multi‐party collaboration. In recent years, advances in artificial intelligence (AI) technology have provided a new paradigm for restructuring the MC model. Large language models (LLMs) and multiagent systems (MASs), with their capabilities for panoramic knowledge integration, multiagent collaboration simulation, and real‐time dynamic optimization, have the potential to support medical information integration and collaborative management [4, 5].

Various models have been used to augment traditional clinical services in both specialty and primary care settings, ranging from medical knowledge enhancement [6, 7] and health document generation [8] to multimodal diagnostic assistance [9, 10] and chronic disease management [11]. The extensive parameters and processing functionalities of intelligent systems have also supported tiered screening‐diagnosis schemes [12], context‐aware triage, and referral coordination [13, 14], facilitating the orderly and efficient flow of information and patient streams across different levels of healthcare institutions. AI‐driven solutions in surgical scheduling [15], financial analysis [16], and health insurance expenditure [17] have shed light on algorithmic administrative operations and evidence‐based regional resource planning. However, there is still ample space to expand LLMs and other models into more diverse tasks, such as assigning billing codes and conversational dialogue [18]. Some consortia (e.g., the Shenzhen Dapeng Medical Group [19]) and health information systems (e.g., the European Clinical Database [20]) have explored frameworks for AI‐driven ecosystems. However, it remains unclear how to systematically design key functional modules that suit consortia's practical demands and synthesize technological pathways to realize intended objectives.

**Figure 1:**
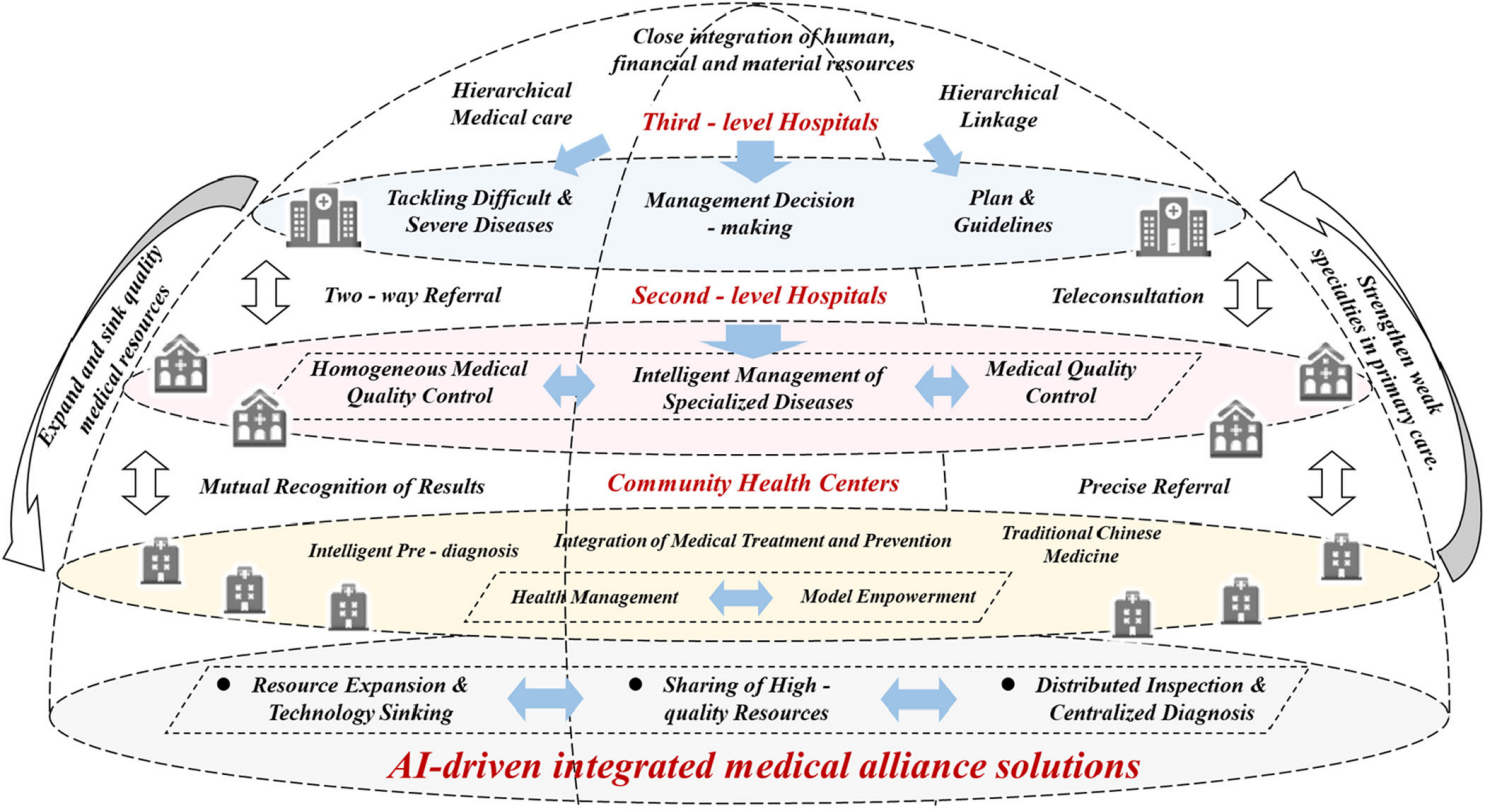
AI‐driven integrated medical alliance solutions.

This study, therefore, aimed to design an AI‐based multiagent collaborative solution framework to integrate the demands of patients, health professionals, and administrators and promote the intelligent transformation of the MC system. Figure 1 shows the specific approach used. The first step was to establish a multidimensional medical knowledge repository as the foundation, systematically integrating heterogeneous data, including medical textbooks, clinical guidelines, and health insurance policies, to form an intelligent decision engine. Second, we constructed a multiagent collaborative architecture of hierarchical triage agents, evidence‐based support agents, and resource scheduling agents to provide customized services for each healthcare actor. Third, we developed integrated functional modules such as intelligent consultation, precise literature retrieval, continuous health management, and cross‐institutional administrative analysis. Fourth, we built interactive output strategies and a well‐structured integration platform to perform professional tasks. Finally, we illustrated practice‐oriented applications and explored robust strategies like blockchain tracking systems to address the underlying risks of the implementation.

## 2 Methods: System Architecture and Technical Implementation

The platform uses a hybrid model of distributed deployment with centralized coordination, rather than a purely centralized cloud system. The core coordination module was deployed at the data center of the leading hospital or consortium to support task scheduling and model management. Member institutions retained their local data and operated lightweight agent modules for preprocessing and inference, balancing collaboration with data security.

The system then applied a multiagent architecture to enable multi‐party collaboration, integrating the needs of patients, doctors, and administrators to ensure smooth information flow and task coordination. The system was structured into five layers—data fusion, perception, decision‐making, presentation, and application—each supported by mechanisms such as retrieval‐augmented generation (RAG)‐based retrieval, federated learning, and large‐model reasoning. This transforms heterogeneous demands within the consortium into a systematic and adaptive workflow.

### 2.1 System Architecture Design

The system followed a classic coordinator–worker pattern and was built around a planning agent, a group of tool agents, and a main agent. The planning agent handles overall task planning and coordination. After receiving a user request, it reviews requirements, drafts an execution plan, and assigns work to appropriate subagents. Tool agents fall into two groups, general agents and specialized agents, carrying out routine medical work and custom specialty tasks. Each subagent works on assignments independently, drawing on its own features and abilities, and then reports back to the main agent. The main agent gathers all the feedback and produces a complete answer. This layered layout breaks a complex job into a number of smaller jobs and improves efficiency and response speed by using a parallel work approach. Formally, the overall workflow can be decomposed into three mathematical stages:

(1) **Task Decomposition by the Planning Agent**
$$𝒫(Q)=\{T_{1},T_{2},\ldots ,T_{n}\},\;T_{i}=f_{\text{plan}}(Q,c_{i})$$
  where Q is the user query, and each $T_{i}$ is a subtask generated according to contextual constraint $c_{i}$.
(2) **Independent Execution by Tool Agents**
$$R_{i}=A_{i}(T_{i},D_{i})=f_{{\text{agent}}_{i}}(T_{i},D_{i}),$$
  where subagent $A_{i}$ processes task $T_{i}$ using its domain knowledge or dataset $D_{i}$, producing intermediate result $R_{i}$.
(3) **Aggregation and Optimization by the Main Agent**

$$R_{\text{final}}=F_{\text{main}}\;(R_{1},R_{2},\ldots ,R_{n})=\text{arg}\max_{r\in 𝒞}U\;(r|Q,\{R_{i}\}),$$

where the main agent integrates results and selects the final answer $R_{\text{final}}$ from candidate space 𝒞 by maximizing a utility function U(⋅) that measures coherence, completeness, and medical relevance.

A unified communication scheme was used. Clear interfaces and protocols let agents exchange information smoothly and can run either synchronously or asynchronously as the task and schedule demand. For data handling, the system applies multi‐level security to keep patient information private and compliant. All sensitive data are encrypted and protected by access control rules that limit their visibility. Data can also be identified to guard patient privacy. The system supports federated learning, making it possible for different institutions to share data and train models together without central storage or transfer. This also improves security.

**Figure 2:**
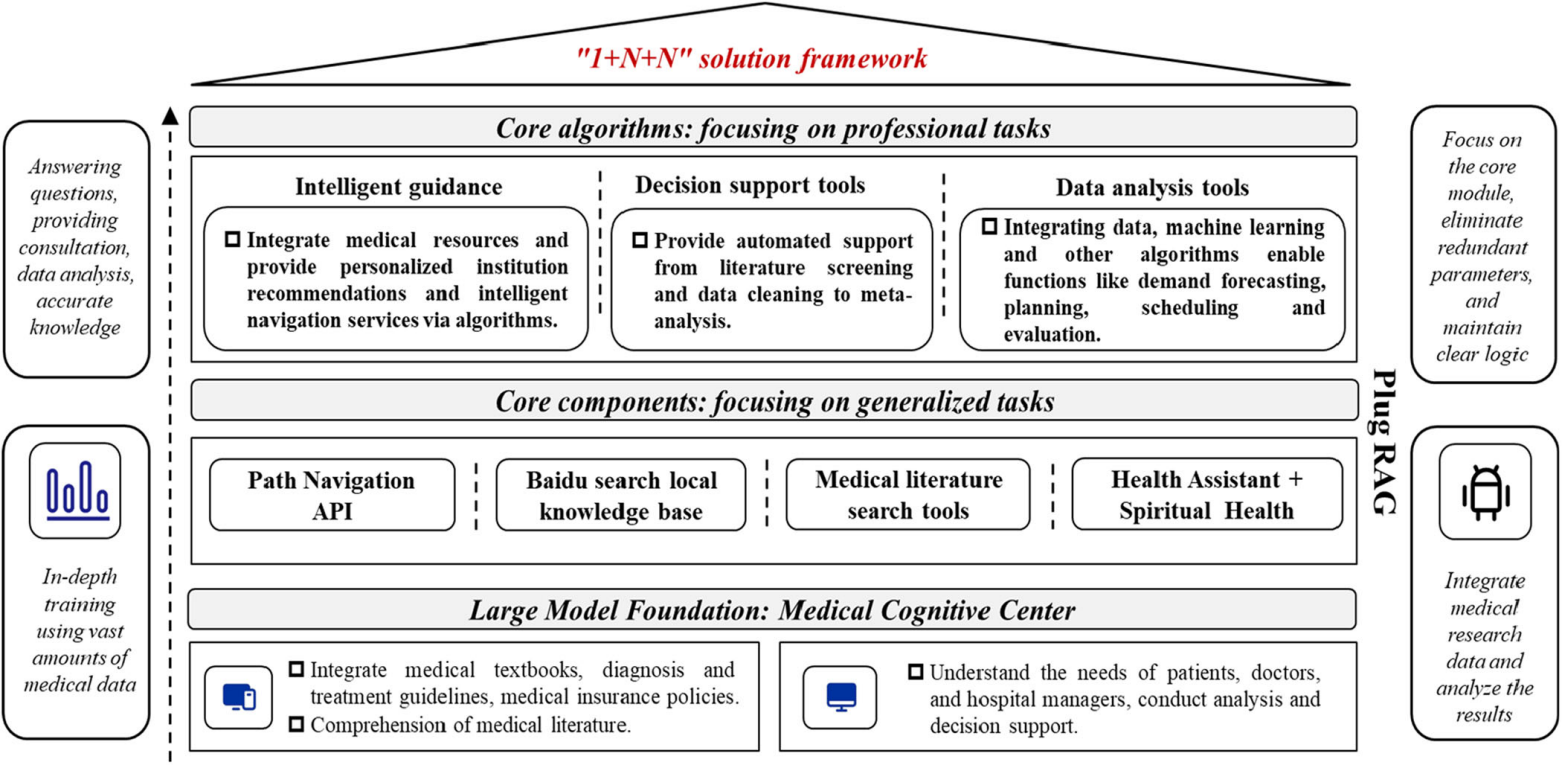
“1 + N + N” solution framework.

To keep pace with the changing needs of medical alliances, the system is built in modules. Each functional block is largely independent and simple to extend. Modules can be loaded or removed on demand and can mesh smoothly with outside systems such as Hospital Information Systems (HISs) to keep information flowing and shared. The system architecture design is shown in Figure 2.

### 2.2 Core Functional Modules

The patient agent improves the visit through smart inquiry, health check, and guidance. After a patient enters their symptoms and condition, the agent, powered by an LLM and a health assistant, creates a health report and diagnostic advice, and then identifies the right hospitals and departments, providing intelligent triage. Live health data tracking supports personal health management, helps patients handle chronic illness, and promotes prevention and early action.

$$O_{\text{pat}}=f_{\text{pat}}\;(S_{\text{sym}},\;X_{\text{HIS}},\;K_{\text{fusion}}),$$

where $S_{\text{sym}}$ is patient symptom input, $X_{\text{HIS}}$ is structured clinical data, and $K_{\text{fusion}}$ is the unified multi‐source knowledge base. The output $O_{\text{pat}}$ is diagnostic advice and personalized guidance.

The doctor agent brings together medical history, test reports, and the latest papers to offer accurate treatment plans, tackle complicated cases, and raise work speed and decision quality. Built‐in literature search and review allow doctors to access new findings and care guides. The agent also writes medical notes, runs quality checks, flags drug and insurance problems, keeps data correct, and reduces errors.

$$O_{\text{doc}}=f_{\text{doc}}\;(X_{\text{HIS}},\;R_{\text{lit}},\;K_{\text{fusion}}),$$

where $R_{\text{lit}}$ is evidence retrieved from external literature (via RAG), which complements HIS data and internal knowledge. The output $O_{\text{doc}}$ is optimized treatment recommendations and clinical documentation.

The management agent studies running data in real time to fine‐tune resource use and workflow. It gives scheduling tips to avoid waste or crowding. The agent forecasts patient flow, bed use, and doctor shifts, then drafts resource plans with quality watch and risk alerts, spots potential problems, and suggests fixes. This provides solid support for managers.

$$O_{\text{mgr}}=f_{\text{mgr}}\;(X_{\text{ops}},\;K_{\text{fusion}}),$$

where $X_{\text{ops}}$ is an operational signal such as patient flow, bed occupancy, and doctor schedules. The output $O_{\text{mgr}}$ is optimized resource allocation and risk alerts for hospital management.

### 2.3 Technical Implementation

#### 2.3.1 Data Layer

**Figure 3:**
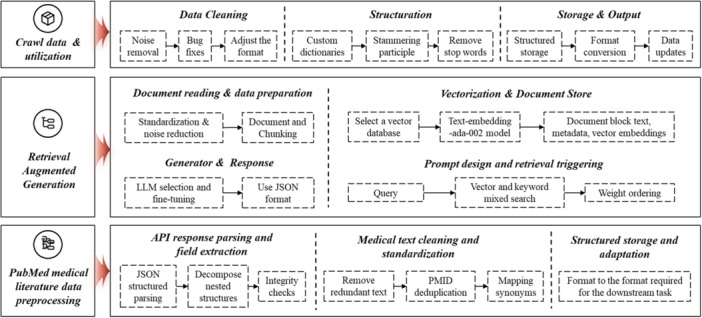
Technical processing pathway.

The data layer merges information from the HIS, medical literature databases, and a local RAG knowledge base for analysis. Using standard APIs, the system pulls patient charts, test results, and treatment histories from the HIS and then carries out data cleaning, noise reduction, and normalization to enable intelligent modules to work with a consistent record set. The detailed process is shown in Figure 3.

$$X_{\text{HIS}}=\text{Norm}\;(\text{Clean}({\text{API}}_{\text{HIS}}({𝒫}_{\text{patient}}))),$$

where ${𝒫}_{\text{patient}}$ denotes raw records, Clean(⋅) removes inconsistency, and Norm(⋅) ensures standard format.

The system also applies RAG to query PubMed in real time, quickly locating the newest studies and clinical guidelines. This increases search efficiency and ensures treatment advice is consistent with the latest research.

$$R_{\text{lit}}=\underset{D_{j}\in {𝒟}_{\text{PubMed}}}{\text{Top}-\text{k}}\langle E(q),E(D_{j})\rangle ,$$

where E(⋅) maps the clinical query q and candidate documents $D_{j}$ into a semantic space.

The layer also contains a local knowledge base, which combines classic evidence‐based resources from both traditional Chinese and Western medicine with internal hospital data. This allows the agent to use both in‐house and public information.

$$K_{\text{fusion}}=\lambda_{1}X_{\text{HIS}}+\lambda_{2}R_{\text{lit}}+\lambda_{3}K_{\text{local}}.$$

This unified representation ensures that later agents can use both evidence‐based and institution‐specific knowledge.

#### 2.3.2 Perception Layer

The perception layer captures information from the outside world and processes it initially. The main agent calls the ERNIE X1 Turbo 32K LLM to handle data transformation and reasoning. When input arrives from a patient, doctor, or administrator, the main agent turns that input into a standardized task description, expressed as:

T=Φ(I)=Norm(Parse(I)),

where I is the raw input, Parse(⋅) extracts its semantic structure, and Norm(⋅) aligns it to a unified schema.

Using this task description, ERNIE performs early‐stage decomposition into subtasks:

$$𝒮={\left\{S_{j}=f_{\text{ERNIE}}\left(T,\theta_{j}\right)\right\}}_{j=1}^{m},$$

where each $S_{j}$ is a subtask such as symptom analysis or resource scheduling. The subtasks are allocated to the most suitable decision agents via a routing mechanism:

$$A^{*}(S_{j})=\text{arg}\max_{A_{k}\in 𝒜}\;\text{Score}(S_{j},A_{k}).$$

This mechanism ensures that each piece of work is consistently matched to the most appropriate agent.

#### 2.3.3 Decision Layer

The decision layer sits at the center of the system. It uses data gathered by the perception layer to work through the information and produce recommendations. To deliver accurate and personal advice, it blends general‐purpose tools with proprietary agents. General tools provide a baseline result, and proprietary agents use patient vitals, history, and previous treatments to enable customization. This cooperative process can be written as:

$$R_{j}^{\text{final}}=\alpha \;f_{\text{gen}}(S_{j},K_{\text{fusion}})+\beta \;f_{\text{prop}}(S_{j},V,H,P,G),\alpha +\beta =1,$$

where $f_{\text{gen}}$ is guideline‐level reasoning, $f_{\text{prop}}$ applies specialized models using patient data (V,H,P) and medical knowledge graphs G, and α,β are dynamic weights that balance generality and personalization.

During diagnosis and therapy, a general tool will run a first pass and list possible options. Proprietary agents then refine the plan to deliver recommendations that fit the patient.

#### 2.3.4 Presentation Layer

After receiving input information, the main agent invokes the LLM for data analysis, combining the results of planning agents and tool agents. It uses the chain of thought (CoT) reasoning mechanism for multistep analysis. In this process, the system incorporates RAG to access external knowledge bases and medical literature in real time, obtaining the latest research findings and guidelines.

This process can be described as a reasoning chain $\left\{c_{t}\right\}$, where each step builds on previous outputs and external retrievals:

$$c_{t+1}=f_{\text{LLM}}\;(c_{t},\{R_{j}^{\text{final}}\},K_{\text{fusion}},{\tilde{R}}_{\text{lit}}),$$

with $\left\{R_{j}^{\text{final}}\right\}$ being the set of refined decisions from the previous layer, $K_{\text{fusion}}$ the unified knowledge base, and ${\tilde{R}}_{\text{lit}}$ on‐demand retrieved evidence.

The final presentation result is then optimized by selecting the clinical utility‐maximizing output from among candidate outputs 𝒪:

$$O=\text{arg}\max_{o\in O}\;U\;(\text{o}\text{ |}\;\{c_{t}\},\{R_{j}^{\text{final}}\},{\tilde{R}}_{\text{lit}}),$$

where U(⋅) evaluates coherence, personalization, and clinical reliability. By integrating external data with internal reasoning processes, the system ensures that it can provide comprehensive, accurate, and patient‐specific decision recommendations.

#### 2.3.5 Application Layer

The application layer primarily serves end users, providing an interactive platform for patients, clinicians, and hospital managers. Patients use intelligent consultation, health management, and other functions through mini‐programs and collaborate with doctor‐side agents to obtain medical services. Doctors interact with the doctor‐side agents through the integrated HIS system to access patient information, literature support, treatment plans, and other assistance, improving work efficiency and decision‐making quality. Hospital managers use the management agents to make decisions about resource scheduling, quality monitoring, and policy implementation.

This mapping of optimized outputs O to role‐specific services can be formalized as

$$O_{\text{role}}=f_{\text{App}}(O|\text{role}\in \{\text{patient},\text{doctor},\text{manager}\}),$$

where the application function $f_{\text{App}}$ adapts the general decision output into actionable recommendations for different user groups. Feedback from real‐world usage is continuously reintegrated into the local knowledge base:

$$K_{\text{local}}^{\left(t+1\right)}=K_{\text{local}}^{\left(t\right)}+\Gamma \;(\text{logs},\text{outcomes}),$$

with Γ(⋅) denoting the update operator that absorbs interaction logs and treatment outcomes.

**Figure 4:**
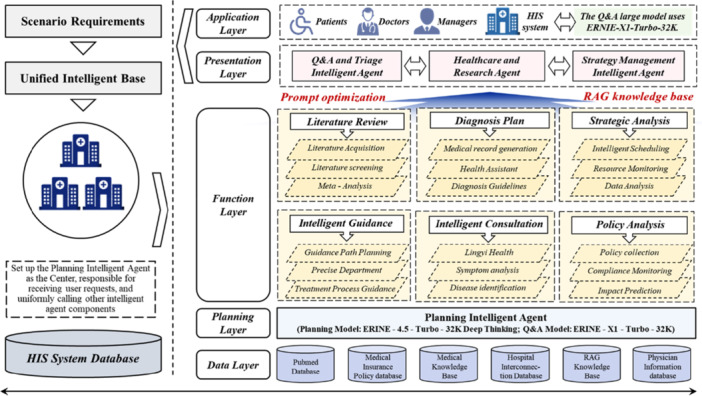
Main functions and interconnections of each layer.

Through this loop of output delivery and feedback integration, the application layer ensures that patients, doctors, and managers can smoothly and accurately access system functions, while the knowledge base evolves dynamically to support long‐term improvements. The primary functions and interconnections of each layer are shown in Figure 4.

## 3 Results: Application Scenarios of AI in Healthcare Systems

The system will be jointly built by consortia, the government, and the National Health Commission. With the reform of medical insurance payment methods, consortia will be prepaid by the medical insurance fund. To ensure service quality and patient health, the MAS for consortia can optimize service processes, manage operations, and implement a hierarchical diagnosis and treatment system. This will reduce management costs and unnecessary medical services and expenses, improving the operational efficiency of consortia. This will support reasonable use of medical insurance funds and medical service improvement, reducing system deployment costs. The deployment of the system creates sustainable economic and health benefits for consortia and stakeholders, while also ensuring the sustainability of the system.

### 3.1 AI Applications for Patients

The patient‐side intelligent assistant works under the operational framework of a close‐knit healthcare network characterized by “primary diagnosis at grassroots level, bidirectional referral, acute and chronic disease differentiation, and vertical integration.” It provides comprehensive health management services through functions such as departmental data integration, precise doctor recommendations, and navigation of treatment pathways.

In the pre‐diagnosis stage, patients can use the intelligent assistant for smart consultations and preliminary diagnoses [21]. After they have input their symptoms, the intelligent assistant can query pre‐established knowledge bases and literature databases to provide scientific and reasonable diagnoses and treatment recommendations. This provides patients with a preliminary understanding of their conditions before seeking medical care. This is designed to improve efficiency and reduce the resource and time costs associated with blind medical consultations.

To support patient consultations, the intelligent assistant recommends hospitals, departments, and doctors by querying information within the healthcare network. It draws on multidimensional factors such as the urgency of the condition and personal preferences (e.g., distance, cost, and doctor ratings). It also plans the most efficient treatment pathway for the patient. Its one‐click registration feature simplifies the medical process, allowing patients to complete registration on a single platform, improving the convenience and smoothness of accessing healthcare resources. This ensures that patients can efficiently obtain medical services and receive professional treatment in a timely manner.

Finally, to support personalized health management, the system can integrate data from wearable devices and home monitoring equipment, and lifestyle information entered by patients, to provide rehabilitation plans that combine both Western and traditional medicine, along with subsequent health management pathways [22]. This closed‐loop system means that patients receive high‐quality treatment within the hospital and continuous, proactive, and personalized health management services outside, delivering an integrated healthcare model that is “health‐centered” [23, 24].

### 3.2 AI Applications for Doctors

The doctor‐side intelligent assistant can integrate fragmented knowledge, data, and capabilities from various levels of medical institutions within the healthcare network into a real‐time, precise, and traceable decision support system. This optimization enhances medical service processes and improves the quality and efficiency of healthcare delivery.

At the clinical decision support level, the AI agent can dynamically generate contextualized treatment recommendations for doctors based on the unified multisource knowledge base and literature database of the healthcare network [25]. The system draws on a diverse set of clinical resources, including the most up‐to‐date evidence‐based care pathways issued by the central hospital, comprehensive literature from databases such as PubMed, regional variations in pharmaceutical formularies, and current diagnostic test data. These sources enable the intelligent assistant to generate tailored treatment recommendations that fit each patient's clinical profile and reflect the latest research.

The doctor‐side agent is also an efficient and user‐friendly tool for medical literature and knowledge retrieval. Clinicians can ask questions based on their research interests or clinical challenges, and the system rapidly queries authoritative sources such as PubMed, returning curated summaries of relevant literature. This functionality streamlines the evidence‐gathering process, helping physicians access high‐quality scientific information more easily and efficiently.

Lastly, the intelligent assistant automates electronic medical record writing through a voice recognition module [26]. It can automatically complete fields such as medical history, allergy history, and physical examination based on templates and knowledge bases, generating initial medical records that comply with the Subjective Objective Assessment Plan (SOAP) format and are standardized to fit the main hospital's documentation. Doctors only need to verify and supplement the generated records to complete the writing process. This functionality significantly reduces physicians' clerical workload and ensures the quality of medical records through standardized templates and knowledge bases. This minimizes record‐writing errors caused by human oversight and improves the completeness and accuracy of the records.

### 3.3 AI Applications for Hospital Management

The management‐side intelligent assistant works within the context of the dual objectives of integrated management of personnel, finance, and materials and multi‐institutional collaboration within the healthcare network. It focuses on transforming operational, quality, and policy data from across the main hospital, branch hospitals, community centers, rehabilitation institutions, and third‐party service providers into real‐time, alertable, and actionable decision directives.

It analyzes operational data and optimizes resource allocation to provide a platform for managers to monitor operational metrics of the hospital in real time. By leveraging the real‐time analytic and alert functions of the intelligent assistant, hospital administrators can promptly identify operational bottlenecks and assess the severity of emerging issues [27, 28]. The assistant also offers targeted recommendations to support timely and effective decision‐making. Administrators can also monitor the allocation of resources across the integrated healthcare system in real time. When patient demand fluctuates among different medical institutions, the system enables managers to issue responsive directives, ensuring the dynamic redistribution of resources within the network to meet evolving clinical needs.

**Figure 5:**
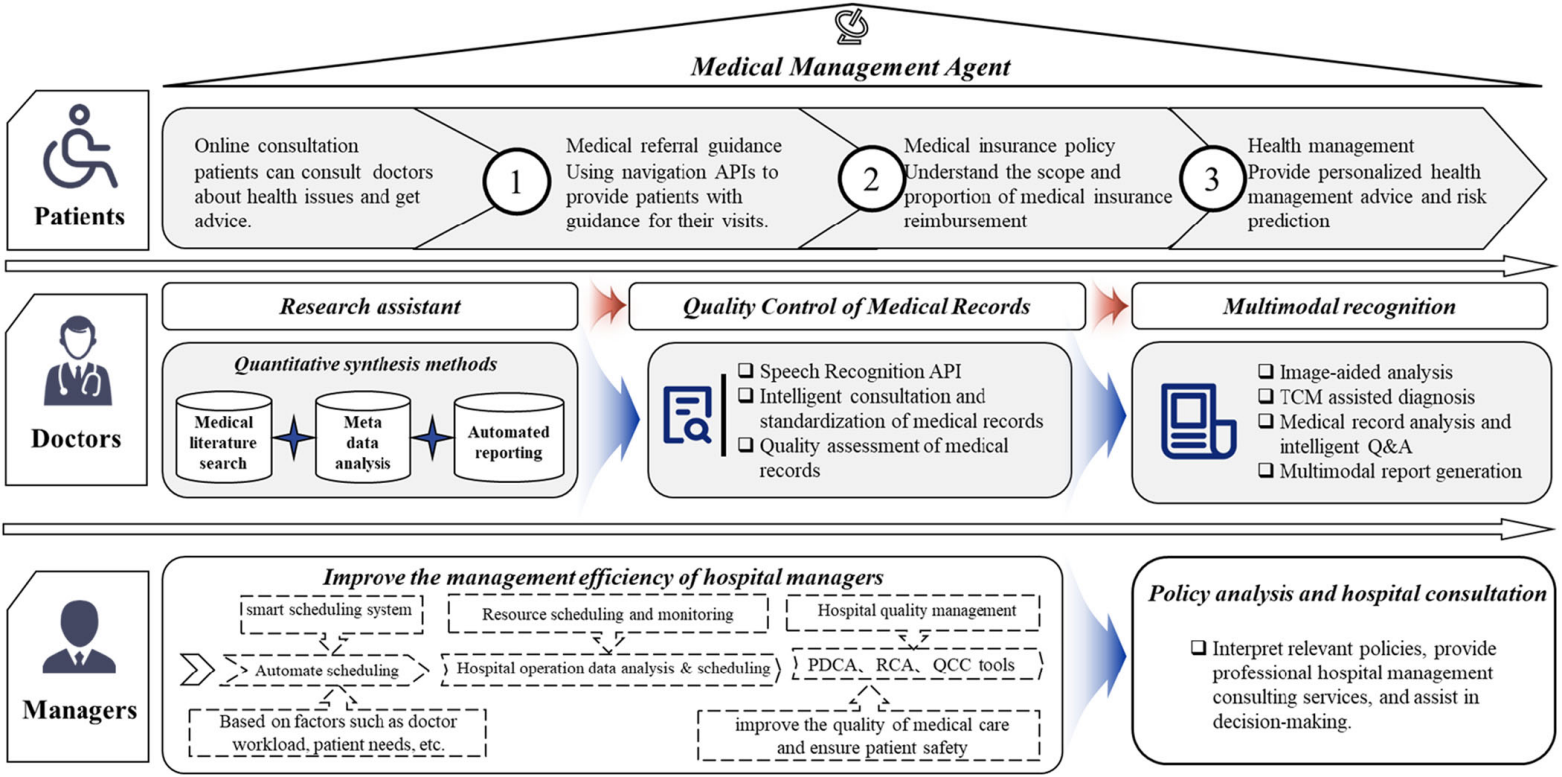
Functionality implementation of three application scenarios.

In quality management and risk prevention, the management‐side intelligent assistant can issue real‐time alerts about workflow anomalies. This helps the healthcare network promptly identify and resolve issues in everyday operations, ensuring the quality and safety of medical services. The application scenario for implementing the functions of medical personnel, management, and patients is shown in Figure 5.

## 4 Discussion: System Advantages, Challenges, and Future Directions

### 4.1 System Advantages and Application Value

This system is based on a multiagent architecture, creating a highly integrated, scalable, and broadly applicable intelligent medical decision support platform that covers a wide range of medical business scenarios. Through parallel processing of general and specialized tasks, the system can flexibly meet various levels of medical needs [29]. The innovation of the system lies primarily in the design of the agent architecture. General‐purpose agents enhance the capabilities of large models in handling medical tasks, improving the efficiency of general task processing. Specialized agents focus on more complex and personalized medical tasks, ensuring that the system can provide highly customized decision support to meet different needs [30, 31, 32]. The use of general‐purpose and domain‐specific agents enables the system to deliver contextually precise decision support, tailored to the specific characteristics of application scenarios and user roles, addressing a diverse and multi‐level range of medical demands [33, 34].

### 4.2 Challenges

The deployment and development of medical intelligent agents can improve the quality of healthcare. However, they also raise challenges related to system reliability, data security, and smooth operation. These challenges include risks during implementation, such as uncertain decision‐making and data privacy concerns [35], and difficulties in development, like inefficient toolchains and cost–performance trade‐offs [36].

During deployment, medical intelligent agents face a major issue: hallucinations in LLMs, where clinical suggestions are based on false or unverified information [37]. These mistakes can lead to wrong diagnoses, unsuitable treatments, wasted resources, and risks to patient safety. For example, using nonstandard data may result in incorrect care plans that interrupt clinical routines. In medical AI systems, data security and privacy remain key concerns. When combining data from multiple sources in the cloud, there is a higher risk of unauthorized access during transfer or storage, which can reduce patient trust [38]. Another major issue is the use of false or unverified data, which may lead to incorrect decisions [39]. If medical agents rely on nonstandard data, they might produce misleading results.

To ensure the responsible use of the AI platform, we propose a multilayered regulatory mechanism, including compliance with Chinese medical device regulations and AI ethical standards, diagnostic suggestions reviewed by medical professionals, multiple data security measures, and the establishment of an ethical review committee. The decision‐making process of the platform should be fully transparent to doctors and patients to ensure that suggestions are understandable and reviewable.

### 4.3 Future Directions

Medical intelligent agents could revolutionize healthcare through targeted advances across multiple domains. In personalized precision medicine, they can leverage individual patient data, such as genetic profiles and lifestyle factors, to develop highly tailored diagnostic and treatment strategies [40]. For automated medical services, enhanced capabilities in intelligent triage and automated report generation can significantly reduce the workload of healthcare professionals, improving operational efficiency. Cross‐regional collaboration through medical consortia also enables agents to facilitate resource sharing and diagnostic expertise, promoting equitable access to high‐quality care [41]. In disease prediction and prevention, agents can analyze large‐scale population health data to identify risks early and deliver proactive, evidence‐based recommendations [42].

By addressing implementation risks through evidence‐based safeguards and robust security measures, and by overcoming development challenges with integrated toolchains and cost‐effective strategies, we can enable medical intelligent agents to become reliable, efficient, and accessible tools. These developments will improve the precision and safety of medical care and help to shift healthcare toward a more personalized, fair, and effective approach [43].

### 4.4 System Implementation and Feasibility Assurance

Specific system implementation and feasibility must be considered to ensure the platform's practical deployment and long‐term sustainability. The platform will be developed on existing open‐source AI frameworks to build core functions, with secondary development tailored to hospital needs. The hospital's Information Center will lead the project, overseeing system architecture, data integration, and operations. Universities and technology enterprises will provide expertise in model fine‐tuning, knowledge graph construction, and privacy‐preserving computation, in a “hospital‐led with external collaboration” model. The Information Center can manage system integration and routine operations, but advanced modules such as large‐model applications and federated learning require support from specialized partners.

To ensure information security, a multilayered strategy will be adopted: localized deployment with federated learning to avoid centralized data leakage; end‐to‐end encryption, hierarchical access control, and de‐identification to secure transmission and storage; and blockchain‐based traceability with third‐party audits to ensure accountability. Through safeguards across technical, institutional, and governance levels, the platform will enable innovation while complying with national data security and privacy regulations.

The platform has been deployed in pilot areas within a single consortium to verify its technical feasibility and practicality, and particularly its stability and efficiency in real‐world applications. This is an early‐stage technical solution, and we have not yet conducted data collection and comparative analysis. After the pilot phase concludes, we will collect and analyze data based on initial feedback to further optimize the platform and assess its effectiveness. We have developed an evaluation plan based on relevant Chinese government policies (such as the “Healthy China 2030” Plan, the Implementation Opinions on Tiered Diagnosis and Treatment, the State Council's Guidelines on Promoting the Construction and Development of Medical Alliances, and the Medical Service Quality Management Measures) to evaluate the platform's effects on healthcare efficiency, diagnostic accuracy, and patient experience. In the future, we will expand the comparative data and real‐world case studies before and after implementation to fully validate the platform's effects.

## 5 Conclusions

This paper presents an intelligent collaboration framework for medical alliances based on a multiagent architecture, integrating RAG knowledge bases, the ERNIE large model, and CoT reasoning mechanisms to systematically optimize healthcare services. The AI platform is expected to facilitate efficient collaboration among patients, doctors, and managers, supporting full‐cycle health management, precise decision support, and dynamic resource scheduling. This should improve the continuity, accuracy, and efficiency of medical services. The platform is currently in the pilot stage, and its real‐world effectiveness has not yet been fully validated, but its architecture and functional potential suggest that future implementation and evaluation data will further substantiate its impact on improving healthcare quality and optimizing resource allocation.

The main theoretical contribution of this study lies in establishing a transparent and reproducible methodology for distributed healthcare intelligence, tailored to the specific needs of medical alliances. By embedding task decomposition, agent cooperation, and knowledge fusion into a formalized multi‐layer structure, the framework provides a generalizable paradigm for intelligent medical collaboration. On the practical side, technical risks such as model hallucination, data security, and cost control can be mitigated through evidence‐chain verification, federated learning, and hybrid cloud deployment.

Looking forward, this framework has potential for expanding personalized precision medicine, cross‐regional MC collaboration, and disease prediction and prevention. This could drive the transformation of healthcare systems toward more intelligent, equitable, and high‐value models. This kind of development will provide critical technological support for achieving long‐term national health strategies such as the “Healthy China” initiative.

## Author Contributions

**Zihao Bian:** conceptualization, methodology, software, data curation, investigation, validation, formal analysis, writing – original draft, writing – review and editing. **Zhiyi Luo:** conceptualization, methodology, software, data curation, investigation, validation, writing – original draft. **Wenhui Zhang:** conceptualization, methodology, software, data curation, investigation, validation, writing – original draft. **Fanyi Kong:** conceptualization, methodology, investigation, validation, formal analysis, supervision, writing – review and editing. **Yiyi Yang:** conceptualization, investigation, writing – original draft, writing – review and editing, formal analysis, supervision. **Yiyang Chen:** investigation, validation, writing – review and editing, writing – original draft, supervision, formal analysis. **Chenjia Liao:** software, validation, investigation, formal analysis, writing – original draft, writing – review and editing. **Ziyi Chen:** software, methodology, writing – review and editing, supervision. **Wei Wang:** writing – original draft, investigation, validation, formal analysis, supervision, methodology, conceptualization. **Wanyun Zhong:** writing – review and editing, validation, formal analysis, supervision, project administration, investigation. **Tuo Li:** supervision, validation, writing – review and editing, formal analysis, project administration. **Nan Wang:** validation, formal analysis, supervision. **Rongfang Zhu:** investigation, validation, formal analysis, supervision. **Gen Li:** writing – original draft, writing – review and editing, supervision, formal analysis, validation, investigation. **Kangwei Shi:** methodology, validation, software, writing – original draft. **Ruizhe Shi:** writing – original draft, methodology, software, investigation, validation. **Zeyu Zhang:** conceptualization, methodology, resources, writing – review and editing, supervision, funding acquisition, project administration. **Zongjiu Zhang:** resources, supervision, formal analysis, project administration, funding acquisition, investigation, validation, visualization, conceptualization.

## Ethics Statement

The authors have nothing to report.

## Consent

The authors have nothing to report.

## Conflicts of Interest

Professor Zongjiu Zhang is a member of the *Health Care Science* Editorial Board. To minimize bias, he was excluded from all editorial decision‐making related to the acceptance of this article for publication. The remaining authors declare no conflicts of interest.

## Data Availability

The data that support the findings of this study are available on request from the corresponding author. The data are not publicly available due to privacy or ethical restrictions.
